# Impacts of the Mindfulness Meditation Mobile App Calm on Undergraduate Students’ Sleep and Emotional State: Pilot Randomized Controlled Trial

**DOI:** 10.2196/66131

**Published:** 2025-06-11

**Authors:** Tovan Lew, Natnaiel M Dubale, Erik Doose, Alex Adenuga, Holly E Bates, Sarah L West

**Affiliations:** 1 Department of Biology, Trent University, 1600 W Bank Drive, Peterborough, ON, K9L 0G2, Canada, 1 705 748 1011 ext 6129; 2 School of Graduate Studies, Trent University, Peterborough, ON, Canada; 3 Department of Kinesiology, Trent University, Peterborough, ON, Canada

**Keywords:** mindfulness, mobile apps, students, mHealth, sleep, emotional state, pilot study, depression, anxiety, stress, university, mobile phone, mobile health

## Abstract

**Background:**

Undergraduate students frequently experience negative emotional states and sleep quality, which is believed to have worsened following the COVID-19 pandemic.

**Objective:**

This study piloted the use of a popular mobile mindfulness app (Calm) as a potential intervention to improve state depression, anxiety, stress, and sleep quality in undergraduate students attending a Canadian university, following the COVID-19 pandemic.

**Methods:**

Undergraduate students were randomly assigned to a control or treatment group and completed a series of 3 questionnaires to evaluate baseline state emotional health (Depression Anxiety Stress Scale 42-Item Version [DASS-42], Perceived Stress Scale 10-Item Version [PSS-10], and Pittsburgh Sleep Quality Index). Treatment group participants were instructed to engage with the fully-automated Calm app’s sleep section for 30 days: 20 minutes daily, 5 days a week, along with an additional 30 minutes of interaction with other app sections each week, resulting in a goal of 130 minutes per week. The control participants were instructed to continue with everyday life and refrain from the use of mindfulness-based apps for 30 days. Following the 30-day treatment period, all participants repeated the 3 questionnaires. The impact of the treatment on all outcomes was examined using linear mixed model analyses. Independent samples *t* tests were used to determine if psychosocial health or sleep scores differed between baseline and follow-up and if differences in such scores were present between the groups.

**Results:**

A total of 80 students met the inclusion criteria and were randomly assigned to the control (n=40) or treatment (n=40) group. One control participant was lost to follow-up and 3 treatment participants discontinued engaging with the Calm app. Both control (n=39) and treatment (n*=*37) groups began with similar demographic, emotional state, and sleep characteristics. Treatment participants engaged with the Calm app’s sleep section for an average of 234 minutes per week; however, 54% (20/37) met the minimum prescribed interaction time across all 4 weeks. Following the 30-day treatment period, compared to the control group, the treatment group’s state anxiety (mean 14, SD 7.4 vs mean 12, SD 7.8; *P*=.002), state stress (DASS-42: mean 20, SD 8.8 vs mean 15, SD 8.5; *P*<.001; PSS-10: mean 22, SD 5.9 vs mean 19, SD 5.9; *P*=.02), and sleep quality (mean 7.7, SD 2.7 vs mean 6.4, SD 3.5; *P*<.001) improved. Posttreatment, state stress and perceived stress severity was lower in the treatment versus control group (DASS-42: *P*=.02; PSS-10: *P*=.03, respectively).

**Conclusions:**

These pilot findings indicate that a mindfulness app may be an effective tool for reducing state anxiety and stress, as well as enhancing sleep quality among undergraduate university students. A larger, randomized controlled trial should confirm these findings.

## Introduction

Poor emotional health is a considerable problem among university students, with current evidence suggesting depression, anxiety, and stress are frequently reported among this population [1-4]. For instance, in a World Health Organization survey of students from 19 universities across 8 countries, 35% of participants met the diagnostic criteria for either a mood, substance-use, or anxiety disorder [1]. Recent systematic reviews suggest the COVID-19 pandemic led to a considerable worsening of psychosocial health among postsecondary students [5,6]. Furthermore, a growing consensus in the literature suggests that, unlike in the general population, postsecondary student mental health has not returned to prepandemic levels [7-15].

Poor sleep quality is another common observance among postsecondary students [16], with conditions such as delayed sleep phase syndrome being 50% more prevalent among postsecondary students compared to the general population [17]. A distinctive combination of social, work, and academic pressures renders this population particularly susceptible to poor sleep quality [18,19]. Sleep disturbances can have enduring effects on mental and physical well-being, having been associated with conditions including cardiometabolic diseases, cancer, and chronic respiratory diseases [20]. Sleep issues have also been linked to decreased academic productivity [21], performance [21], and increased dropout rates in postsecondary students [22].

While bidirectional relationships are believed to exist between each of depression, anxiety, and stress [23-26], a recent meta-analysis of randomized controlled trials does suggest poor sleep plays a causal role in the development of reduced psychosocial health [27]. Accordingly, poor sleep quality may create a cyclic effect wherein worse sleep quality negatively impacts psychosocial health, thereby further complicating the maintenance of healthy sleep habits [23,26,28,29]. As increasing levels of depression, anxiety, and stress have been linked to lower levels of help-seeking intention [30], additional targeted resources aimed at improving state emotional health and sleep quality are needed among postsecondary students.

Mindfulness interventions aim to extend upon the levels of attention and awareness in normal functioning by fostering sustained, nonjudgmental present-moment awareness [31]. These practices, which commonly include focused attention meditation, body scanning, and mindful breathing, aim to mitigate cognitive distractions and promote a deliberate focus on internal and external experiences [31]. Recently, mindfulness meditation has advanced to include mobile applications offering users the convenience of guided practice at their preferred time and location. These resources offer a potential alternative to traditional mental health supports and may address the most common barriers (such as long wait times and confidentiality concerns) to accessing mental health care as reported by a recent random sample of approximately 2000 Canadian students [15]. Current meta-analyses suggest mindfulness-based online or app-based tools are effective in significantly improving both mental health and sleep quality in postsecondary students [32-35]. While reviews generally agree that online and app-based mindfulness tools can significantly reduce anxiety and stress in students, the evidence regarding their impact on depression is mixed [32-34,36]. Moreover, few studies to date have explored the impact of an app-based mindfulness intervention on the critical intersection of emotional well-being and sleep quality in postsecondary students.

Therefore, the following pilot study aimed to explore the impact of a 1-month stand-alone treatment using the mindfulness-based app Calm on state emotional health and sleep quality in a sample of undergraduate students attending a Canadian university post the COVID-19 pandemic. We hypothesized that using the Calm app would improve undergraduate students’ sleep quality and symptoms of state stress, anxiety, and depression relative to a control group. As this was a pilot investigation, we also hypothesized that using the Calm app for the duration of this study would be feasible, as determined by meeting the minimum suggested minutes of app interaction per day.

## Methods

### Study Design and Participants

A pilot nonblinded randomized controlled study was conducted among undergraduate students attending a university in Ontario, Canada. Inclusion criteria were as follows: (1) be 17 years of age or older, (2) be enrolled in full-time undergraduate studies, (3) possess a smartphone or tablet capable of effectively running the Calm app, (4) not be regular users or subscribers of the Calm app, and (5) provide informed consent. Internet literacy was an implicit eligibility requirement. Past or current diagnoses of clinical depression or anxiety were not considered an exclusion criteria for this study. Participant recruitment occurred during 2 periods: between January 31 and February 13, 2023 (winter term), and September 21 and October 13, 2023 (fall term). Participants were recruited on a first-come, first-served basis using an approved script distributed via email to student mailing lists across various academic departments at the university. Word of mouth among students also contributed to participant recruitment.

The Calm app is a smartphone app available from developer Calm.com on iOS (Apple Inc) and Android (Open Handset Alliance [led by Google]) operating systems, that includes a variety of mindfulness features such as guided meditation, sleep stories, breathing exercises, and mindfulness-based stress reduction techniques [37]. The app’s sleep section houses several resources designed to improve sleep quality, such as guided relaxation exercises, sleep stories, and calming soundscapes [37]. The Calm app was selected due to its comprehensive approach and popularity. Study participants were randomized to either the treatment or control group by study personnel via the random allocation functions in Microsoft Excel (version 16.83, Microsoft Corporation). An identical in-person questionnaire was administered to participants by study personnel at 2 time points: before (baseline) and after (follow-up) the 30-day Calm app treatment period.

**Figure 1.**
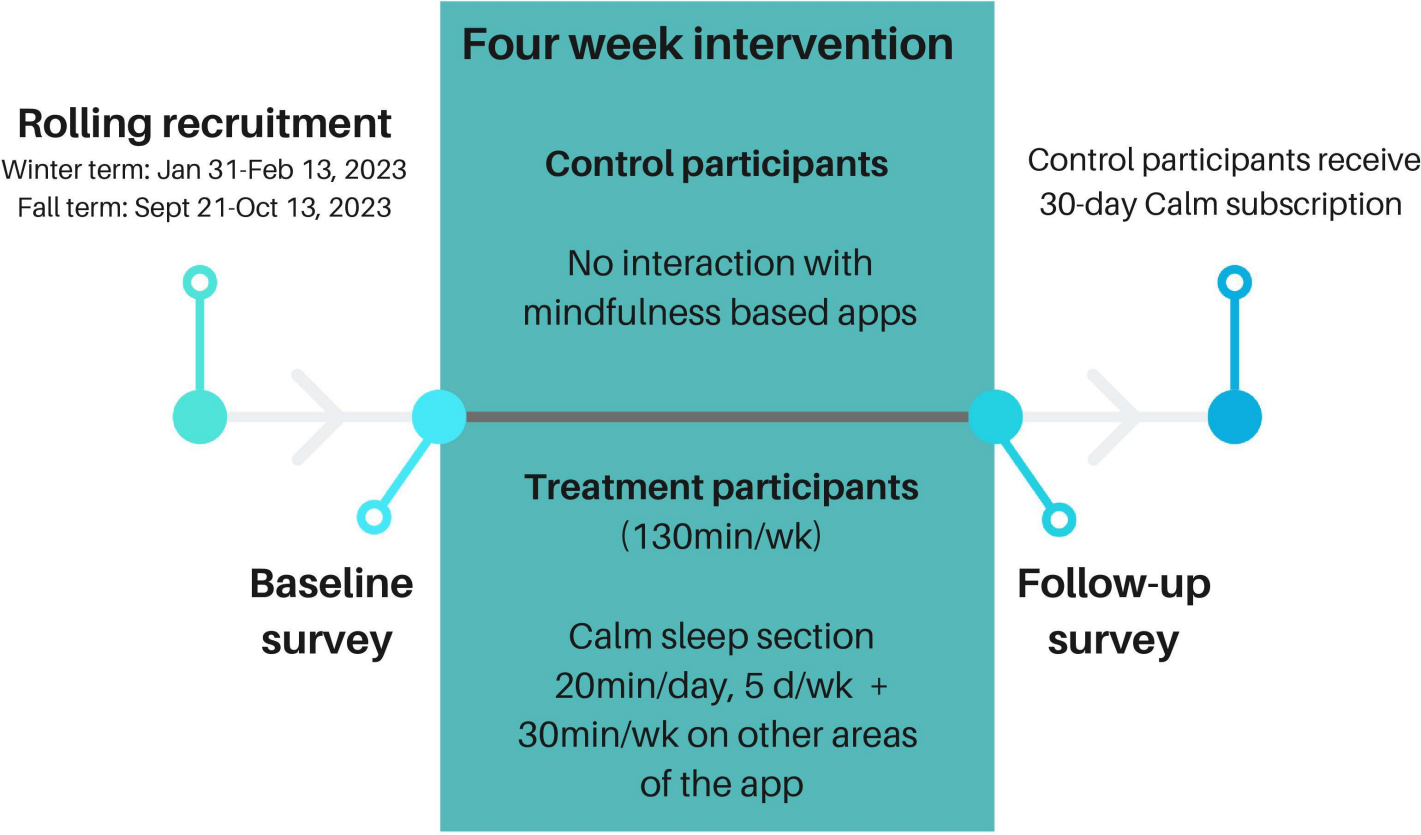
Study design line schematic including recruitment, baseline, intervention, and follow-up.

Participants randomized to the treatment group received 1-month of access to the Calm app, accessible via a unique URL. The 30-day length of intervention was selected for the current pilot study as this was thought to be a realistic, shorter timeframe to allow for an initial assessment of the potential benefits of using the Calm app. The treatment group was instructed to use the app’s sleep section for 30 days: 20 minutes daily, 5 days a week, along with an additional 30 minutes of interaction with other app sections each week, resulting in a total of 130 minutes of app use per week. This amount of time was selected for the intervention as it was thought to be similar to one 30-minute meditation class per day. We advised use for 5 days per week, instead of 7, to provide students with greater flexibility and enhance the probability of sustained engagement. Compliance was tracked using a logbook; participants filled out the total daily time spent using the Calm app during the 30-day treatment period. The control group was instructed to continue with their usual daily routines, refraining from the use of any mindfulness apps such as Calm. After the 30-day study period, and following the completion of the follow-up questionnaires, the control group received free access to the Calm app for 1 month. Figure 1 describes this study timeline.

### Ethical Considerations

This study was reviewed and received approval from Trent University’s Research Ethics Board (28256). Given that this study was a pilot investigation, the protocol was not registered within a clinical trials database. Following ethics approval, interested participants were provided with information about this study via a consent form and were provided with the opportunity to review the form and ask questions about this study to a member of the research team. Written informed consent was obtained from all participants. Each participant was assigned a unique study ID number to ensure privacy and confidentiality. Access to 1 month of the Calm app served as the incentive for study participation, provided to the treatment group during their study interval and the control group for 1 month following their 30-day study interval.

### Questionnaire and Measures

The questionnaire consisted of 4 components including a demographic questionnaire which collected age, gender, year of study, and program of study. Components 2 to 5 consisted of validated self-assessed psychological tools: the DASS-42 (Depression Anxiety Stress Scale 42-Item Version) [38], the Pittsburgh Sleep Quality Index (PSQI) [39], and the Perceived Stress Scale 10-Item Version (PSS-10) [40]. The DASS-42 is a validated tool consisting of 3 sections, each containing 14 subgrouped questions assessing state depression, anxiety, and stress [41-43] and has been used to assess emotional states among postsecondary students previously [44-46]. The DASS-42 produces 3 scores ranging from 0 to 42, each corresponding to either state depression, anxiety, or stress [47]. Such scores can be categorized as normal to extremely severe according to the following scale for anxiety, stress, and depression, respectively: normal (0‐7, 0‐14, and 0‐9), mild (8-9, 15-18, and 10-13), moderate (10-14, 19-25, and 14-20), severe (15-19, 26-33, and 21-27), extremely severe (≥20, ≥34, and ≥28) [47].

The PSQI is a 10-item self-report that evaluates sleep over the past month on 7 domains, producing a global sleep quality score. Higher scores correspond to poorer sleep quality [39]. Such domains include sleep quality, latency, duration, efficiency, disturbance, medication use, and daytime dysfunction [39]. The PSQI has been validated through previous studies [48,49] and has been used to examine sleep in undergraduate students [50-52]. The PSS-10 assesses self-perceived stress over the past month using 10 questions on a 5-point Likert scale [40]. Stress levels can then be categorized as follows: scores from 0 to 13 indicate low stress, scores from 14 to 26 indicate moderate stress, and scores from 27 to 40 indicate high stress [53]. The PSS-10 has been validated among university students [54], and a systematic review of studies assessing the PSS-10’s validity supports its use [55].

### Statistical Analyses

Demographic data was analyzed using descriptive statistics. Two-sample *t* test with equal variances was used to compare mean age between treatment and control groups, and Pearson chi-squared tests were used to determine if significant differences in categorical demographic variables existed between these groups. Independent *t* tests were used to determine if baseline and posttreatment scores differed significantly for each of the 5 measures (DASS-depression, DASS-anxiety, DASS-stress, PSS-stress, and PSQI-sleep) between treatment and control groups. Linear mixed models were computed for each of this study’s outcomes, examining the interaction between time point (baseline or follow-up) and study group (treatment or control). Effect sizes (Cohen *d*) were calculated for linear mixed model outcomes, with thresholds defined as large (*d*≥0.80), medium (0.50≤*d*<0.80), and small (0.20≤*d*<0.50). Statistical significance was set at *P*<.05. The term of data collection (winter 2023 or fall 2023) was also included in the models to examine if the term of study had an impact on the treatment and study outcomes. Statistical analyses were conducted in R (version 4.3.2, R Foundation for Statistical Computing), and the following packages were used for analyses: lme4, lmerTest, and effsize. Figures were generated using Microsoft Excel.

## Results

### Participant Exclusion and Demographic Characteristics

**Figure 2.**
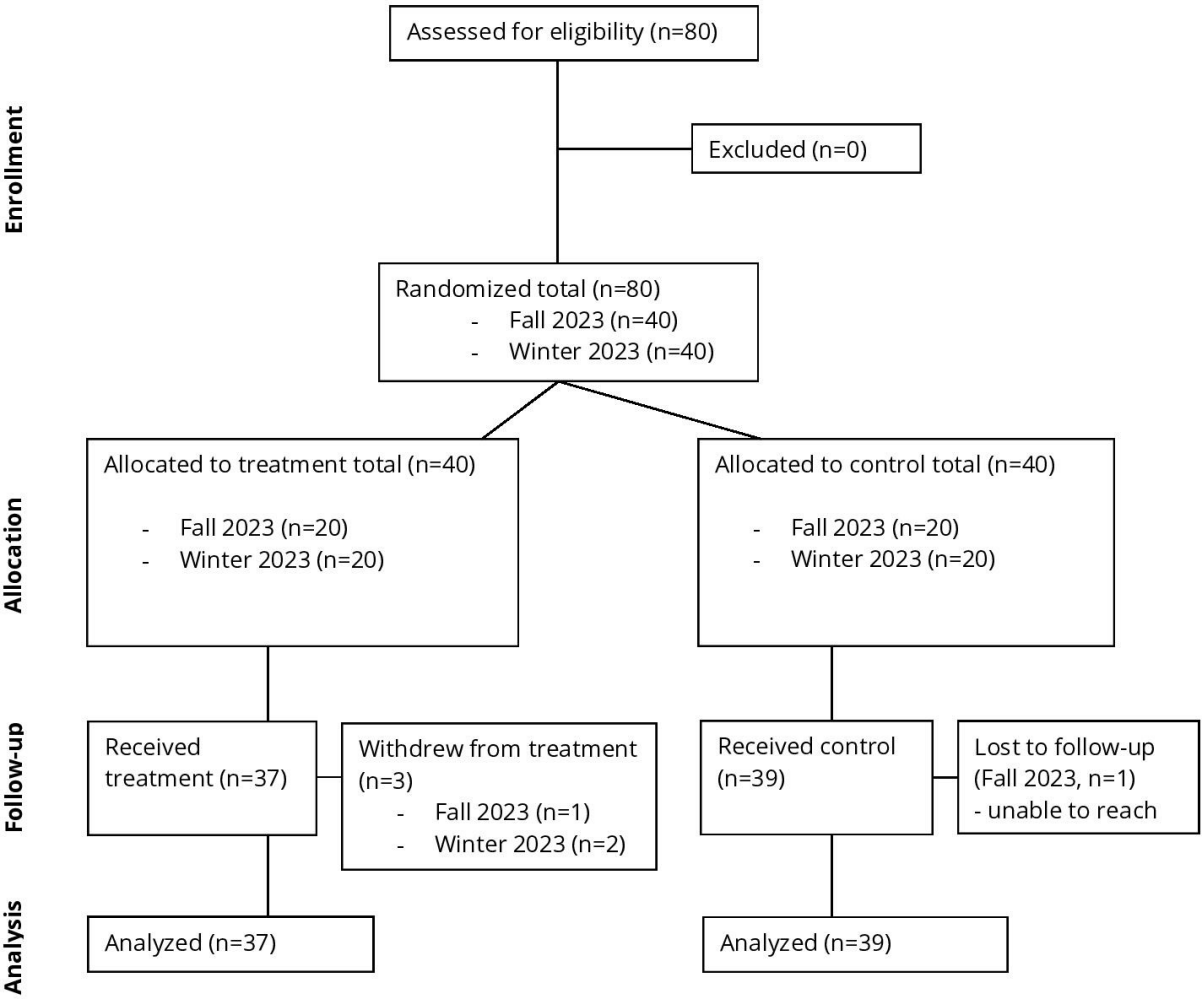
CONSORT flow diagram of participants’ eligibility, enrollment, group allocation, treatment, and data analyzed in this pilot study. CONSORT: Consolidated Standards of Reporting Trials.

A total of 80 participants, equally distributed between fall and winter recruitment terms, were randomly assigned to either the treatment (n=40) or control (n*=*40) group. One participant during the fall recruitment term and 2 participants during the winter term withdrew from this study, leading to their exclusion from the treatment group. Additionally, one participant was excluded from the control group due to loss at follow-up during the fall data collection period. Therefore, data from 76 undergraduate students (38 from each collection period; 39 in the control, and 37 in the treatment) were included in the analysis (Figure 2).

Table 1 presents demographic data by study group. The mean age of participants did not differ significantly (*P=*.27) between the 2 groups (mean 20.6, SD 3.1 and mean 21.8, SD 5.8 years in the control and treatment groups, respectively). Most participants identified as female, including 82% (32/76) and 81% (30/76) of participants in the control and treatment groups, respectively (*P=*.80). Students’ year of study ranged from year 1 to year 5+. There was also an equal distribution of students by year of study (*P=*.59) and program of study (*P=*.21) between the control and treatment groups.

**Table 1.** Demographic characteristics of participants, comparing the control and treatment groups (N=76). All programs are bachelor’s degrees.

Characteristic	Control group (n=39)	Treatment group (n=37)	*P* value
Age (years), mean (SD)	20.64 (3.09)	21.81 (5.76)	.27
Gender, n (%)			.80
Female	32 (82)	30 (81)	
Male	5 (13)	6 (16)	
Gender nonconforming	2 (5)	1 (3)	
Year of study, n (%)			.59
First year	11 (28)	10 (27)	
Second year	8 (21)	5 (14)	
Third year	11 (28)	12 (32)	
Fourth year	9 (23)	8 (22)	
Fifth year or higher	0 (0)	2 (5)	
Program of study, n (%)			.21
BA^a^	2 (5)	4 (11)	
BAS^b^	2 (5)	1 (3)	
BESS^c^	1 (3)	0 (0)	
BSc^d^	18 (46)	24 (65)	
BScFS^e^	10 (26)	3 (8)	
BScKin^f^	0 (0)	2 (5)	
BScN^g^	4 (10)	2 (5)	
BSW^h^	2 (5)	1 (3)	

a BA: Bachelor of Arts.

b BAS: Bachelor of Arts and Science.

c BESS: Bachelor of Environment Science or Studies.

d BSc: Bachelor of Science.

e BScFS: Bachelor of Science in Forensic Science.

f BScKin: Bachelor of Science in Kinesiology.

g BScN: Bachelor of Science in Nursing.

h BSW: Bachelor of Social Work.

### State Depression, Anxiety, and Stress

#### Baseline

Table 2 presents pretreatment outcome data. The mean state depression scores pretreatment were similar in the control and treatment groups (*P*=.58) and represented moderate severity state depression at baseline in both groups. The mean state anxiety scores at baseline were also similar between the control and treatment groups (*P*=.11), however, scores represented moderate-severity state anxiety in the control group and severe state anxiety in the treatment group. Baseline mean DASS-42 (*P*=.85) and PSS-10 (*P*=.10) scores were comparable between groups, both indicating moderate stress severity. Overall, 26%‐44% (10/39 to 17/39) of those in the control group experienced severe to extremely severe levels of state depression, anxiety, and stress. Comparatively, 24%‐54% (9/37 to 20/37) of participants in the treatment group reported similar levels.

**Table 2.** Mean baseline state emotional health scores, comparing the control and treatment groups (N=76).

Measure	Control group (n=39)		Treatment group (n=37)		*P* value
Mean (SD)	Score category	Mean (SD)	Score category
DASS^a^-depression	15.41 (11.05)	Moderate	14.08 (9.91)	Moderate	.58
DASS-anxiety	14 (7.02)	Moderate	16.81 (8.27)	Severe	.11
DASS-stress	21.38 (8.4)	Moderate	21.73 (7.09)	Moderate	.85
PSS^b^ -stress	22.15 (6.88)	Moderate	22.16 (6.39)	Moderate	.10

a DASS: Depression Anxiety Stress Scale 42-Item Version.

b PSS: Perceived Stress Scale 10-Item Version.

#### Pre- Versus Posttreatment

**Figure 3.**
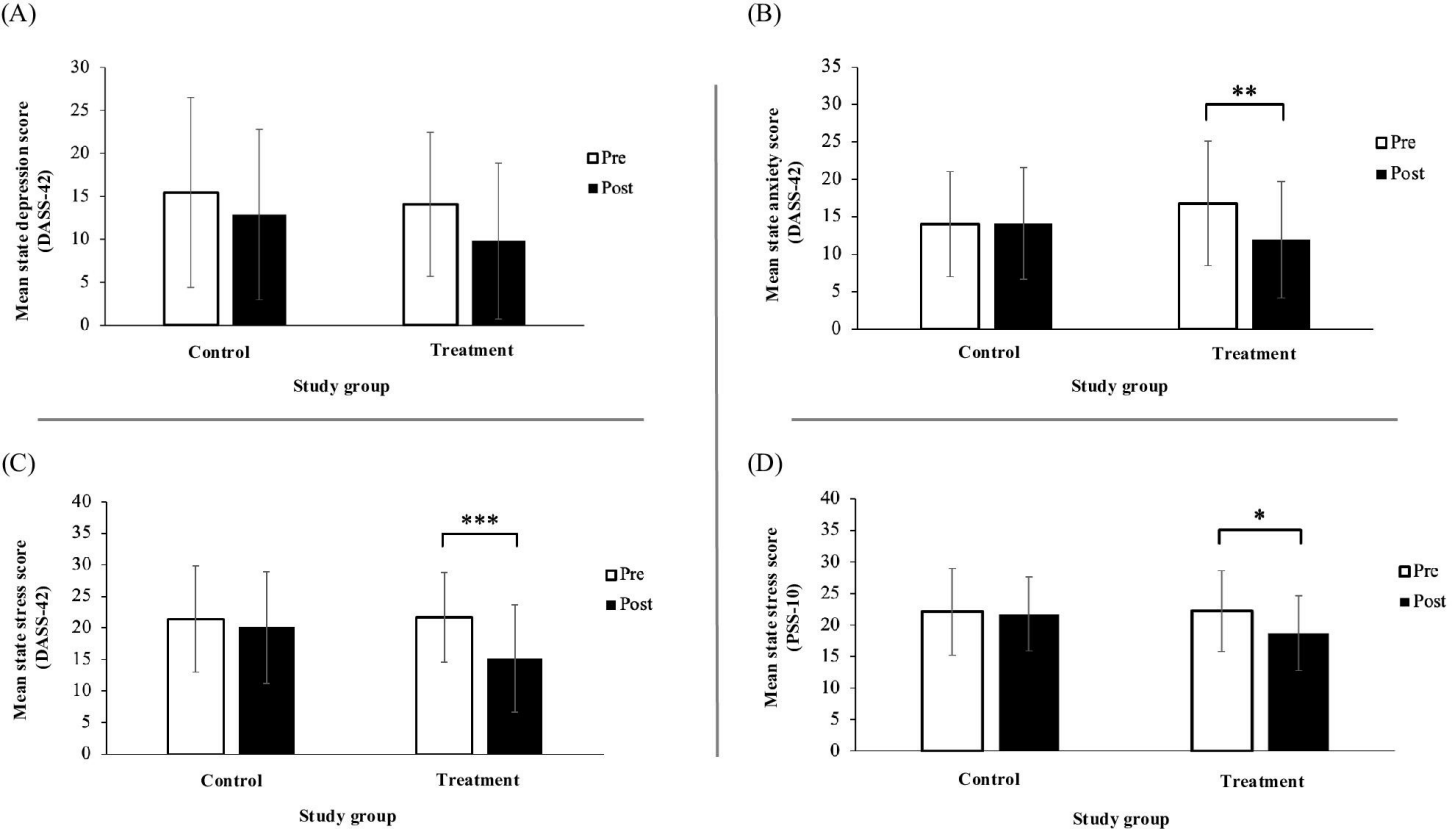
Depression, anxiety, and stress scores among the control (n=39) and treatment (n=37) groups pre- and posttreatment with the Calm app. Peaks of the bar columns represent the mean score, and error bars represent the SD. Statistical analysis by linear mixed model. (**A**) Mean DASS-42 state depression. (**B**) Mean DASS-42 state anxiety. (**C**) Mean DASS-42 state stress. (**D**) Mean PSS-10 state stress. Mean state anxiety and state stress measured by the DASS-42 and PSS-10 decreased significantly in the treatment group following 30 days of treatment. There were no changes in any of the state emotional health outcomes in the control group between baseline and 30 days later. DASS-42: Depression Anxiety Stress Scale-42; PSS-10: Perceived Stress Scale-10. *P<.05, **P<.01, ***P<.001.

Table 3 summarizes the interaction of time point and study group for all study outcomes. State depression showed no significant interaction (*P*=.37; Figure 3A), and Cohen *d* effect size of the treatment group’s changes in state depression was small (*d*=0.4, 95% CI 0.16 to 0.73). The interaction of time point and study group for state anxiety demonstrated a significant interaction (*P=*.002; Figure 3B), with a medium effect size within the treatment group (*d*=0.6, 95% CI 0.26-0.95). The interaction of time point and study group for stress also showed a significant interaction for both the DASS-42 stress score (*P*<.001; Figure 3C) and PSS-10 stress score (*P*=.02; Figure 3D). The treatment group’s change in stress had a large effect size for DASS-42 stress score (*d*=0.81, 95% CI 0.53-1.10) and a medium effect size for PSS-10 stress score (*d*=0.57, 95% CI 0.24-0.89).

There was no impact on outcome measures between the 2023 winter term dataset and the 2023 fall term dataset (*P*>.16; Table 4).

**Table 3.** Linear mixed model of the interaction of time point × study group for state emotional health outcomes.

Variables	Precontrol (n=39), mean (SD)	Postcontrol (n=39), mean (SD)	Control effect size (95% CI)	Pretreatment (n=37), mean (SD)		Posttreatment (n=37), mean (SD)	Treatment effect size (95% CI)	*t* value (*df*)	*P* value
DASS^a^-depression	15.4 (11.05)	12.9 (8.37)	0.25 (-0.03 to 0.53)	14.1 (9.91)		9.81 (9.08)	0.45 (0.16 to 0.73)	–0.9 (74)	.37
DASS-anxiety	14 (7.02)	14.1 (7.44)	–0.004 (–0.23 to 0.22)	16.8 (8.28)		11.95 (7.82)	0.6 (0.26 to 0.95)	–3.24 (74)	.002
DASS-stress	21.4 (8.4)	20.1 (8.84)	0.1 (–0.05 to 0.34)	21.7 (7.09)		15.2 (8.54)	0.81 (0.53 to 1.1)	–4.05 (74)	<.001
PSS^b^-stress	22.12 (6.88)	21.74 (5.92)	0.06 (–0.19 to 0.32)	22.2 (6.4)		18.7 (5.91)	0.57 (0.24 to 0.89)	–2.47 (74)	.02

a DASS: Depression Anxiety Stress Scale 42-Item Version.

b PSS: Perceived Stress Scale 10-Item Version.

**Table 4.** Linear mixed model of interaction of fall and winter term data collection × all outcome measures.

Outcome measure	*t* value (*df*)	*P* value
DASS^a^-depression	-0.24 (72)	.81
DASS-anxiety	-1.43 (72)	.16
DASS-stress	0.86 (72)	.39
PSQI^b^	-0.23 (72)	.82
PSS^c^-stress	-0.33 (72)	.75

a DASS: Depression Anxiety Stress Scale 42-Item Version.

b PSQI: Pittsburgh Sleep Quality Index.

c PSS: Perceived Stress Scale 10-Item Version.

#### Posttreatment Follow-Up

Mean follow-up state emotional health scores are summarized in Table 5. The mean state depression follow-up scores were similar (*P=*.13) for the control and treatment groups, both of which were in the mild category. The mean follow-up state anxiety scores were similar between groups and corresponded to a moderate level of state anxiety in both groups. The mean DASS-42 stress scores at follow-up were mean 20.13 (SD 8.84) and mean 15.22 (SD 8.54) in the control and treatment groups, respectively, corresponding to a moderate severity in the control group and a mild severity in the treatment group. The mean PSS-10 stress scores at follow-up were mean 21.74 (SD 5.92) and mean 18.68 (SD 5.91) in the control and treatment groups, respectively, falling in the moderate stress category [53]. Both the DASS-42 and PSS-10 stress scores were higher in the control versus treatment group at study end (*P*=.02 and *P*=.03, respectively).

**Table 5.** Mean scores of state emotional health in control compared to treatment groups at posttreatment or 30 day follow-up.

Variables	Control group (n=39)			Treatment group (n=37)		*P* value
	Mean (SD)	Score category		Mean (SD)	Score category	
DASS^a^-depression	12.87 (8.37)	Mild		9.81 (9.08)	Mild	.13
DASS-anxiety	14.03 (7.44)	Moderate		11.95 (7.84)	Moderate	.24
DASS-stress	20.13 (8.84)	Moderate		15.22 (8.54)	Mild	.02
PSS^b^-stress	21.74 (5.92)	Moderate		18.68 (5.91)	Moderate	.03

a DASS: Depression Anxiety Stress Scale 42-Item Version.

b PSS: Perceived Stress Scale 10-Item Version.

### Sleep Quality

#### Baseline

Baseline sleep quality scores were not significantly different (*P=*.14) between the control (mean 8.18, SD 3.15) and treatment (mean 9.46, SD 4.26) groups. This represents a categorization of poor sleep quality in both groups as indicated by a score greater than 5 [39]. Most participants in both groups displayed poor sleep quality, with 82% (32/39) in the control group and 84% (31/37) in the treatment group falling into this category. Over three-quarters of the treatment (28/37, 76%) and control (31/39, 79%) groups exhibited insomnia according to previously established cutoffs.

#### Pre- Versus Posttreatment

**Figure 4.**
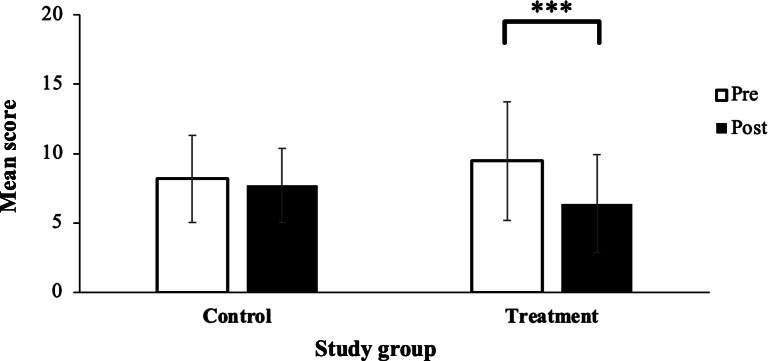
Mean PSQI scores among the control (n=39) and treatment (n=37) groups pre- or posttreatment with the Calm app. Peaks of the bar columns represent the mean score, and error bars represent the SD. Statistical analysis by linear mixed mode. Mean PSQI score decreased (improved) significantly in the treatment group following 30 days of treatment with the Calm app. There were no changes in PSQI score in the control group between baseline and 30 days later. PSQI: Pittsburgh Sleep Quality Index. ***P<.001.

The interaction of time point and study group for sleep quality showed a significant interaction (*P*<.001; Figure 4), with a medium effect size within the treatment group (*d*=0.78, 95% CI 0.45 to 1.10).

#### Posttreatment Follow-Up

The mean sleep quality scores at follow-up were mean 7.69 (SD 2.66) and mean 6.38 (SD 3.53) in the control and treatment groups, respectively. This represents a categorization of poor sleep quality in both groups [39]. An independent sample *t* test found no difference between the control and treatment sleep quality scores at follow-up (*P=*.07).

### Adherence

Over half (20/37, 54.1%) of treatment group participants completed the minimum requested interaction time with Calm (130 min/wk) across all 4 weeks. Differences in treatment group adherence (how many weeks the minimum interaction time was met) between participants recruited during the fall ($\bar{x}$=3.3, SD=1.2) and winter ($\bar{x}$=2.6, SD=1.7) terms were not significant (*P=*.14), with a small effect size (*d*=0.48). The total average time that treatment group participants interacted with the Calm app’s sleep section was 663.6 minutes in the fall group and 1208.7 minutes in the winter group (*P=*.18), representing a small effect size (*d*=0.4). Importantly, the average minimum number of minutes fall participants spent using the sleep section (144.1 min/wk) was similar to the winter participants (106.4 min/wk; *P*=.1), with a medium effect size (*d*=0.55). The most used sections of the app outside of sleep were music (10/33, 30%), daily calm (5/33, 15%), and soundscapes (5/33, 15%).

## Discussion

### Principal Findings

This pilot study found that following a 30-day treatment period with the Calm app, undergraduate students assigned to the treatment group displayed significant reductions in state anxiety and stress severity as well as improvements in sleep quality relative to the control group. While both groups had similar outcome measures at baseline, state stress levels were significantly lower in the treatment group compared to the control group at follow-up. This represents a decrease in state stress levels that are similar to the lower range found in worldwide representative samples pre–COVID-19 pandemic [56-62]. Moreover, the treatment group experienced a 16% reduction in the prevalence of poor sleep quality. Such improvements in state anxiety, stress, and sleep quality, despite only 54% adherence to the full-prescribed use of the Calm app, are particularly encouraging. These findings suggest that even partial engagement with mindfulness interventions can lead to meaningful benefits. They also indicate that the app’s effects may be more substantial or easier to attain than expected, potentially enhancing its feasibility and appeal among university students.

### State Emotional Health in Undergraduate Students

Baseline findings from our sample of undergraduate students indicate that more than one-third of participants experienced severely heightened levels of state depression, anxiety, or stress. This is higher than the worldwide representative scores of state depression, anxiety, and stress among university students documented in the literature. For instance, studies conducted in countries including the United States, Saudi Arabia, Ethiopia, Spain, Turkey, Pakistan, and Malaysia have reported severe or extremely severe prevalence rates of depression in university student samples ranging from 9.7% to 27%, state anxiety from 15% to 41.4%, and stress from 5% to 34% [56-62]. These studies, however, were conducted before the COVID-19 pandemic. Since then, several reports have indicated a decline in psychosocial health among undergraduate students, attributed to factors such as social isolation [63], decreased physical activity [64], and uncertainty about the future [65] as a result of the COVID-19 pandemic. Indeed, in a study of students at the same postsecondary institution, our research group found that over half of the students surveyed displayed moderate-to-extremely severe state anxiety, stress, or depression, and that state anxiety and stress worsened in students assessed in 2021 compared to 2020 [7].

Digital mindfulness-based interventions have been shown to improve a variety of psychosocial health indicators in student populations, including depression, stress, anxiety, and general mental well-being [66-70]. Like the findings of this study, in a systematic review and meta-analysis, Chen et al [33] reported that mobile-device-based mindfulness interventions appeared to significantly improve stress and anxiety but not depression among postsecondary students. In contrast, Gong et al [34], Alrashdi et al [36], and Zuo et al [35] found that online mindfulness interventions improved anxiety, stress, as well as depression among university students. Thus, the impact of such mindfulness interventions on depressive symptoms in this population remains unclear. Nevertheless, given that online and app-based meditation tools have shown capacity to improve anxiety and stress severity [32-34,36], the improvements in state anxiety and stress that we observed following Calm app use may be related to the collective content and structure of mindfulness-based apps rather than being attributable specifically to the Calm app itself. This is promising as there may be the potential for developing independent and accessible mobile resources that consider the unique needs of specific postsecondary populations, which can be integrated into the current framework of mental health support available to university students. Nevertheless, further studies are needed to reinforce relationships demonstrated in the extant literature.

### Sleep Quality in Undergraduate Students

At baseline, over 80% of the control and treatment group participants exhibited poor sleep quality (indicated by a PSQI global score >5) [39], with a mean PSQI global score of 8.2 and 9.5 in the control and treatment groups, respectively. Over three-quarters of all participants exhibited insomnia (indicated by a PSQI global score ≥6) [50]. A systematic review of 23 studies conducted before the COVID-19 pandemic revealed that undergraduate students typically exhibited poor sleep, with a mean PSQI global score of 6.3 among the studies [71]. This suggests participants in our sample displayed worse baseline sleep quality than what has been reported in the prepandemic literature. Namely, participants displayed insufficient sleep duration (<7 hours of sleep per night), prolonged sleep onset, increased sleep disturbances (such as restlessness), heightened reliance on sleep medication, and increased sleep-related daytime dysfunction. The presence of elevated state depression, anxiety, and stress at baseline in our population may aid in accounting for the poor sleep quality, because evidence suggests that sleep and mental health influence each other bidirectionally [23-26,71]. Experiencing depression, anxiety, and stress during the day may lead to delayed sleep onset and sleep disruption [71]. Poor sleep may then impair one’s ability to cope with daytime stressors, and increase maladaptive emotional regulation, further exacerbating the experience of negative emotional states and poor sleep and psychosocial health over time [26,28,29,71]. Ultimately, such relationships highlight the value of interventions that simultaneously target psychosocial health and sleep quality among university students.

Previous research on the impact of mobile mindfulness-based interventions on sleep has yielded comparable results to this study. A recent meta-analysis suggests online mindfulness-based interventions can significantly improve sleep quality among postsecondary students [35]. In 1 study involving adults with sleep disturbances, the Calm app was found to significantly reduce fatigue, daytime sleepiness, and presleep arousal compared to controls [72]. Similarly, physician assistant students using the Happier app showed marked improvements in sleep impairment relative to the control group [73], and a study with university students using the Calm app revealed a negative correlation between overall mindfulness and sleep disturbance [74].

Mobile mindfulness interventions may have a multifaceted impact on student well-being, positively influencing both emotional state health and sleep quality. This finding is particularly notable given the complex and interrelated challenges faced by university students, who frequently experience heightened levels of stress, anxiety, and disrupted sleep. A dual impact is especially valuable in an academic context, where students often encounter difficulties in accessing or committing to traditional mental health resources. Therefore, the integration of mobile mindfulness interventions could offer a promising avenue for enhancing overall student well-being and academic performance, thus contributing to a more holistic approach to mental health support within higher education settings.

### Limitations and Future Directions

Our findings should be considered in the context of certain limitations. As our sample consisted of students exclusively from a single university campus in Canada, caution should be applied when generalizing our findings to the broader undergraduate population. Participants were not evenly distributed across demographic categories; there was unequal representation among genders, years of study, and programs of study. The timing of data collection in the fall and winter may have also introduced confounding variables, notably examination periods and seasonal affective disorder (SAD). These potential confounders were not controlled, as the periods in which students are at risk of being impacted by such factors comprise most of the fall and winter terms (September to April of each year). Thus, this was impractical to control in a pilot study. Nevertheless, as these factors have been found to impact undergraduate psychosocial health [75-78], future studies should aim to determine the influence of examination-related stress and SAD on the efficacy of mobile psychosocial health interventions among students. Given the pilot nature of this study, recruitment occurred only during the fall and winter terms (and not the summer term, which occurs from May to August of each year) to maximize the number of potential recruits, as the percentage of full-time students who are taking on-campus classes during the summer term is lower. This study may have also lacked sufficient power due to a small sample size, which may have limited the ability to detect smaller, potentially meaningful differences in app usage patterns, such as the increased time spent in specific sections of the app by the winter group. This could suggest that the positive return on invested in-app time within certain app sections diminishes beyond a minimum threshold. Future studies should aim to recruit larger cohorts during the fall, winter, and summer sessions who are demographically representative of the broader undergraduate population, to aid in better understanding the influence of SAD and other nuances of such mindfulness interventions.

Treatment group adherence must also be considered. Only 54% (20/37) of treatment group participants indicated that they completed the minimum prescribed weekly interaction time with the Calm app. Although this self-reported moderate level of adherence was evidently adequate in impacting psychosocial health and sleep quality, it may have negatively impacted the strength of associations between the treatment and outcome measures. This level of engagement is consistent with previous findings; a systematic review of web- and app-based mindfulness interventions across the general population, including university student cohorts, reported a mean adherence rate of 56% (SD 15%), based on the authors’ established definitions of compliance [79]. Strategies to improve adherence, such as text reminders, have been positively received among university students [80], with program reminders being associated with increased adherence [79]. Without a detailed analysis of the control group’s measured engagement with alternative coping strategies (nonadherence) or placebo treatment, it remains unclear whether these improvements can be attributed solely to the current treatment. As well, we did not consider whether participants had previous experience with mindfulness app usage; this may have impacted participants’ expectations, and future studies should consider accounting for this. If feasible, obtaining access to screen time spent on the app itself, while ensuring confidentiality, would offer a more direct quantification of adherence. Future studies may benefit from implementing engagement strategies such as integrated daily reminders and an active control group.

Given the use of self-reported questionnaires, it must also be noted that self-report biases, including social desirability bias and recall bias, may have impacted our results [81]. This is particularly relevant for the PSQI and PSS-10, which require participants to retrospectively report their sleep habits and feelings of stress over the preceding month [82]. Additionally, variability in bedtimes and wake-up times complicates the accurate reporting of typical sleep patterns and durations [82,83]. The use of a social desirability scale can improve the validity of self-reports in future investigations [81]. Finally, as this is a pilot study with a relatively small sample size, this research is limited by the potential for bias due to incomplete data, as an intent-to-treat analysis was not performed. The small sample size itself also reduces the generalizability of the findings. These results should be validated through larger-scale preregistered clinical trials to confirm their robustness.

### Conclusions

The use of the Calm app over a 4-week period resulted in a significant reduction in state anxiety and stress, while also improving the sleep quality in full-time undergraduate students attending a Canadian university post-COVID-19 pandemic. A moderate level of treatment adherence (54%) suggests that even partial engagement with a mindfulness-based mobile app may lead to improvements in state emotional health and sleep quality. Future studies should explore the effects of mindfulness-based apps across larger samples while implementing interventions during summer terms. Direct quantification methods of treatment adherence may also improve our understanding of these relationships. This study highlights the potential value of mindfulness-based apps as a resource to support both the emotional state and sleep quality of undergraduate students.

## Supplementary material

10.2196/66131 Checklist 1 CONSORT-eHEALTH checklist (V 1.6.1).

## Abbreviations

DASS-42: Depression Anxiety Stress Scale 42-Item Version
PSQI: Pittsburgh Sleep Quality Index
PSS-10: Perceived Stress Scales 10-Item Version
SAD: seasonal affective disorder
